# Preparation and Characterization of Theophylline Controlled Release Matrix System Incorporating Poloxamer 407, Stearyl Alcohol, and Hydroxypropyl Methylcellulose: A Novel Formulation and Development Study

**DOI:** 10.3390/polym16050643

**Published:** 2024-02-27

**Authors:** Molham Sakkal, Mosab Arafat, Priya Yuvaraju, Rami Beiram, Salahdein AbuRuz

**Affiliations:** 1 College of Pharmacy, Al Ain University, Al Ain P.O. Box 64141, United Arab Emirates; molham.sakkal@gmail.com (M.S.); mosab.arafat@aau.ac.ae (M.A.); 2 Department of Pharmacology and Therapeutics, College of Medicine and Health Sciences, United Arab Emirates University, Al Ain P.O. Box 17666, United Arab Emirates; 3 Department of Biopharmaceutics and Clinical Pharmacy, School of Pharmacy, The University of Jordan, Amman 11942, Jordan

**Keywords:** controlled-release matrix system, differential scanning calorimetry, fourier transform infrared spectroscopy, hydroxypropyl methylcellulose, in vitro dissolution, poloxamer 407, scanning electron microscopy, stearyl alcohol, thermogravimetric analysis, theophylline, X-ray diffraction

## Abstract

Background: Theophylline (THN), a bronchodilator with potential applications in emerging conditions like COVID-19, requires a controlled-release delivery system due to its narrow therapeutic range and short half-life. This need is particularly crucial as some existing formulations demonstrate impaired functionality. This study aims to develop a new 12-h controlled-release matrix system (CRMS) in the form of a capsule to optimize dosing intervals. Methods: CRMSs were developed using varying proportions of poloxamer 407 (P-407), stearyl alcohol (STA), and hydroxypropyl methylcellulose (HPMC) through the fusion technique. Their in vitro dissolution profiles were then compared with an FDA-approved THN drug across different pH media. The candidate formulation underwent characterization using X-ray diffraction, scanning electron microscopy, Fourier transform infrared spectroscopy, differential scanning calorimetry, and thermogravimetric analysis. Additionally, a comprehensive stability study was conducted. Results: In vitro studies showed that adjusting the concentrations of excipients effectively controlled drug release. Notably, the CRMS formulation 15 (CRMS-F15), which was composed of 30% P-407, 30% STA, and 10% HPMC, closely matched the 12 h controlled-release profile of an FDA-approved drug across various pH media. Characterization techniques verified the successful dispersion of the drug within the matrix. Furthermore, CRMS-F15 maintained a consistent controlled drug release and demonstrated stability under a range of storage conditions. Conclusions: The newly developed CRMS-F15 achieved a 12 h controlled release, comparable to its FDA-approved counterpart.

## 1. Introduction 

**Figure 1:**
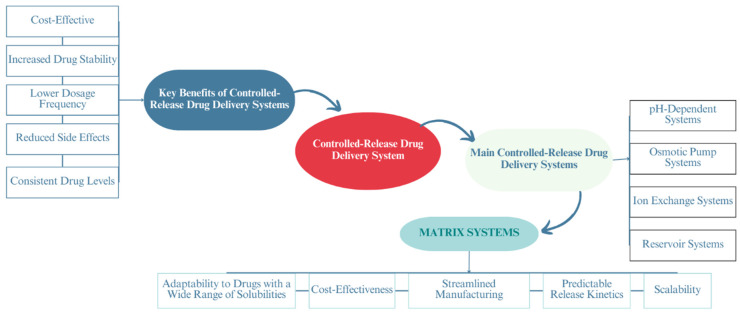
Overview of controlled-release drug delivery systems and characteristics of matrix systems.

Controlled-release drug delivery systems (CDDSs) have significantly transformed the pharmaceutical field. These systems are designed to administer medication in a regulated way over a prolonged period, effectively minimizing the need for frequent dosing [1]. This can improve patient adherence to the medication and overall treatment outcomes [2]. Various additional benefits can be obtained from developing CDDSs, as shown in Figure 1. Various types of medication require the utilization of CDDSs: for instance, drugs with a short half-life, such as furosemide [3], oxcarbazepine [4], and metoprolol [5], as well as medications with a narrow therapeutic window like theophylline monohydrate (THN) [6,7], lithium [8], and phenytoin [9]. However, the development of new CDDSs faces a range of challenges that must be addressed.

New controlled-release (CR) compounds require sufficient stability within the gastrointestinal tract (GIT) while releasing the drug molecules at predetermined intervals. Several medications have been recalled from the market for not meeting dissolution specifications, such as metformin CR tablets [10], Adderall CR tablets [11], indapamide CR tablets [12], and metoprolol succinate CR tablets [11,13]. The frequent recalls of CR medications and the importance of CDDSs in the pharmaceutical industry highlight the ongoing potential for advancements in CDDS development [4,14,15,16]. Thus, the primary objective of this research was to develop new CDDSs suitable for Biopharmaceutical Classification System (BCS) Class I medications, utilizing a minimal number of excipients and a convenient preparation method.

The model drug for this study was THN. THN was selected due to the reported challenges in maintaining controlled drug release functionality in some THN CR formulations [17,18]. Additionally, delivering THN in a CR system is preferred due to its rapid absorption, narrow therapeutic window (10 to 20 mg/mL), and short half-life (7 to 9 h) [6,7]. THN is mainly used for chronic obstructive pulmonary disease (COPD) and asthma [6,19]. Additionally, recent research has shown some potential new pharmacological benefits of THN, for instance, in managing COVID-19 [20] and post-tubercular lung illnesses [21], and as an effective alternative to permanent pacemaker implantation [22]. 

The matrix system stands out among the different developed CDDSs due to its simplicity in manufacturing, cost-effectiveness, and predictable drug release kinetics among other different features, as demonstrated in Figure 1 [3]. In a matrix system, the active pharmaceutical ingredient (API) is uniformly dispersed within a polymeric matrix, effectively controlling the drug release rate [23]. There is a wide range of excipients used in the production of a matrix system. The selection of the matrix polymer depends on the desired release profile, method of preparation, and API characteristics, as well as on the various properties of the excipients [24,25,26,27]. 

In this study, three excipients were selected to develop a new controlled-release matrix system (CRMS). These chosen excipients were poloxamer-407 (P-407), hydroxypropyl methylcellulose (HPMC), and stearyl alcohol (STA). These excipients were chosen based on their physicochemical properties and their effectiveness in retarding drug release according to different research studies [28,29,30,31]. For instance, an in situ gel delivery system combining P-407 and methylcellulose prolonged drug release compared to administering the drug freely [32]. Another study developed an intragastric floating tablet using HPMC and polylactic acid (PLA) that controlled drug release for 24 h [2]. Similarly, STA and HPMC in a CRMS extended the release of sarpogrelate HCL for 24 h [25].

P-407 is extensively utilized in the creation of drug delivery systems (DDSs) due to its various properties. It is known to be non-toxic [33,34], exhibits excellent compatibility [28,33], and has a high capacity to solubilize various medications [33,34]. The ability of P-407 to control drug release is attributed to the amphiphilic nature of poloxamers. This contributes to the formation of a gel around the formulation, which retards the rapid release of the drug molecules [1,31,35,36].

HPMC has a wide range of applications, for instance, as a thickener, emulsifier, and stabilizer [37,38], owing to its compatibility, excellent safety profile, and stability [29]. Moreover, the utilization of HPMC to control drug release is attributed to its capacity to form a gel upon contact with water, establishing a dynamic barrier that controls the drug release rate, as has been reported in the literature [31,39].

STA has diverse applications that are attributed to its unique characteristics, including its emollient properties, viscosity enhancement, pH stability, and non-toxicity [30,40]. Additionally, STA in a CR formulation has the ability to form a hydrophobic domain that hinders drug diffusion and extends release duration [40].

The current study aimed to develop and evaluate a novel matrix-based controlled-release system tailored for THN, a BCS Class 1 drug. Utilizing the fusion method, the system incorporates P-407, STA, and HPMC, and is encapsulated in a size 00 capsule. 

In this study, several formulations with varying polymer ratios and types were prepared. These were assessed using an in vitro dissolution apparatus in media with different incubated pH levels (1.2, 4.5, and 7.5) to simulate varying gastrointestinal environments. Additionally, drug release kinetics were determined to elucidate the drug’s release mechanism from the matrix system. The selected formulation was comprehensively characterized through X-ray diffraction (XRD), scanning electron microscopy (SEM), Fourier transform infrared (FTIR) analysis, differential scanning calorimetry (DSC), and thermogravimetric analysis (TGA). Stability studies were also conducted, involving the exposure of the formulations to various temperatures and humidity levels over a period of 72 h. Additionally, their stability under constant conditions was assessed over a storage period of three months.

## 2. Materials and Methodology 

### 2.1. Materials

THN and P-407 were purchased from Sigma Aldrich (St. Louis, MO, USA). STA and HPMC were ordered from Thermo-Fisher Scientific (Kandel, Germany). Theophylline^®^ CR (300 mg) branded tablets (Napp, Cambridge Science Park, Cambridge, UK) were acquired from a local retail pharmacy (Abu Dhabi, United Arab Emirates). Hydrochloric acid (37%) was acquired from Eurolab (Surrey, UK). Sodium hydroxide pellets were obtained from Surechem Products Ltd. (Suffolk, UK). Sodium phosphate monobasic monohydrate was obtained from VWR Life Science AMRESCO (Radnor, PA, USA).

### 2.2. Methodology

#### 2.2.1. Preparation of a Controlled-Release Matrix System Containing Theophylline 

Four series of polymer matrix systems were prepared using the heat fusion method, each containing a consistent amount of the drug molecule, THN, and varying proportions of the excipients: P-407, HPMC, and STA. This approach aimed to assess the individual and combined effects of these excipients on modulating THN release. All formulations contained 300 mg of THN, matching the therapeutic dose of the reference product. The methods used in preparing these series are detailed in the subsequent sections.

##### Preparation of the First Series of Controlled-Release Matrix Systems: Incorporating THN with P-407

In Series 1, four formulations (F1 to F4) were developed with increasing P-407 concentrations by 10% increments, as detailed in Table 1. These formulations had varying ratios of THN to P-407: (3:2), (1:1), (2:3), and (3:7) for F1, F2, F3, and F4, respectively. To ensure homogeneity, P-407 was initially melted and maintained at 60 °C, then stirred at 250 RPM for 20 min. Subsequently, THN was incorporated into the melted P-407, with continuous stirring at the same temperature (60 °C) for an additional 30 min. The homogeneous gel produced was poured into hard gelatin capsules: size 0 for F1 to F3, and size 00 for F4. The capsules were then left to solidify at room temperature for 15 min. Post solidification, they were refrigerated at 8 °C for preservation and future analysis [31,41,42,43].

##### Preparation of the Second Series of Controlled-Release Matrix Systems: Combining THN, P-407, and STA

In Series 2, all formulations (F5 to F8) consistently contained 300 mg of THN and 450 mg of P-407, as outlined in Table 2. The STA concentration in these formulations increased incrementally, with percentages of 3%, 9%, 17%, and 25% for F5, F6, F7, and F8, respectively. This resulted in THN–P-407–STA ratios of (39:58:3), (36:55:9), (33:50:17), and (30:45:25) for the respective formulations. The preparation process involved stirring P-407 at 250 RPM in a 60 °C water bath. After 20 min, STA was added and stirred for an additional 20 min. Subsequently, THN was incorporated and stirred for 30 min to ensure a homogeneous mixture. The final matrices were then encased in size-00 hard gelatin capsules and left to solidify at room temperature for 15 min, before refrigeration at 8 °C for preservation [31,41,42,43].

##### Preparation of the Third Series of Controlled-Release Matrix Systems: Incorporating THN, P-407, and HPMC

In Series 3, the methodology and ingredient proportions were consistent with those of Series 2, with HPMC replacing STA, as detailed in Table 3. The THN–P-407–HPMC ratios for formulations F9, F10, F11, and F12 were (39:58:3), (36:55:9), (33:50:17), and (30:45:25), respectively [31,41,42,43].

##### Preparation of the Fourth Series of Controlled-Release Matrix Systems: Integrating THN, P-407, STA, and HPMC

In Series 4, consistent with Series 2 and 3, the amounts of THN and P-407 were maintained. However, this series uniquely combined STA and HPMC in varying proportions, detailed in Table 4. The THN–P-407–HPMC–STA ratios for formulations F13, F14, F15, and F16 were (30:30:20:20), (30:30:15:25), (30:30:10:30), and (30:30:5:35), respectively. The preparation process involved melting P-407 at 60 °C for 20 min at 250 RPM, followed by adding STA and HPMC, and stirring for another 20 min. THN was then incorporated and stirred for an additional 30 min to achieve a homogeneous blend. This gel mixture was filled into size-00 gelatin capsules, allowed to solidify at room temperature, and subsequently stored at 8 °C in a refrigerator. To ensure reliability of the results and controlled experimental conditions, all series were prepared within 24 h of assessment, adhering to methodologies of previous studies [43,44,45].

#### 2.2.2. Drug Content Determination

The drug contents for the developed formulations were analyzed to verify the precise drug quantity. Every sample was ground into a fine powder and then dissolved in 900 mL of distilled water. Subsequently, a 1 mL portion of this solution was further diluted with 10 mL of distilled water. The diluted sample was filtered using 0.22 μm syringe nylon filters and analyzed for drug content using a T70 UV/VIS spectrometer (PG Instruments Ltd., Lutterworth, LE17 5FB, UK) at a wavelength of 271 nm. This experiment was conducted in six replicates.

#### 2.2.3. Calibration Curve 

The branded THN tablet was ground into a fine powder using a mortar and pestle. A stock solution of 0.33 mg/mL was then prepared by dissolving the powder in 100 mL of distilled water, stirring at 250 RPM for an hour, and gradually adding more water to reach a total volume of 900 mL. After achieving complete dissolution, this stock solution was diluted to concentrations of 33, 16.5, 8.25, 4.12, 2.06, and 1.03125 µg/mL. UV spectrophotometry was used to measure the absorbance of each dilution, resulting in values of 1.57, 0.83, 0.41, 0.22, 0.11, and 0.06. These values were plotted on a calibration curve, with the dilution concentrations on the x-axis and absorbance on the y-axis. The high regression factor (r^2^) of 0.9991 indicates strong linearity. This experiment was conducted in six replicates [41].

#### 2.2.4. In Vitro Dissolution Studies

The Dis 8000 dissolution apparatus (Copley Scientific, Nottingham, UK) was used to evaluate the drug release from the formulations. The test maintained a rotation speed of 100 RPM and a temperature of 37.5 ± 0.5 °C. All formulations were tested in 900 mL of distilled water as the dissolution medium. Additionally, to assess the drug release profiles under simulated GIT conditions, the formulations were tested in media of three different pH levels: 1.2, 4.5, and 7.5, each prepared to a volume of 900 mL following USP guidelines. The different pH media were prepared and adjusted using hydrochloric acid (37%), sodium hydroxide pellets, and sodium phosphate monobasic monohydrate for pH adjustment [41]. 

Sampling was conducted at specified intervals (0 h to 12 h, hourly) using the DissoFract automated sampler (Copley Scientific, Nottingham, UK), withdrawing 5 mL from each vessel. To maintain constant dissolution conditions, an equal volume of media was replenished after each sample withdrawal, keeping the vessel volume at 900 mL. Each sample was filtered through 0.22 μm syringe nylon filters, and 1 mL of the filtered sample was diluted with 10 mL of distilled water for drug concentration analysis. UV spectrophotometry with a 1 cm quartz cuvette and a UV detector at 271 nm wavelength was used for this analysis. A total of six samples were tested for each time point to ensure statistical reliability. The established calibration curve facilitated the determination of sample concentrations. For each formulation, release profiles in various pH environments were plotted, positioning concentration on the x-axis and the corresponding absorbance on the y-axis [29]. A dissolution profile comparison between the candidate formulation and the reference THN product was conducted using the fit factors demonstrated in the Moore–Flanner model, utilizing two metrics: *f*1 and *f*2. An *f*1 value of 0 signifies a perfect match between the test and reference dissolution profiles, with increasing values indicating greater disparity. Conversely, the *f*2 score ranges from 0 to 100, where 100 indicates identical profiles, with the score decreasing as differences grow [46,47].

The candidate formulation, demonstrating a release profile comparable to the branded drug with extended drug release up to 12 h, was selected for further assessment. This formulation, identified as controlled-release matrix system formulation 15 (CRMS-F15), was then subjected to detailed characterization and stability assessments, as outlined in the forthcoming sections of the methodology.

#### 2.2.5. Release Kinetic Model Evaluation 

The drug release mechanisms of CRMS-F15 and the branded drug were investigated by utilizing various mathematical models. The data derived from the dissolution release profiles were charted across various kinetic models, including the zero-order, first-order, Hixson–Crowell, Higuchi, and Korsmeyer–Peppas models [31,48]. 

#### 2.2.6. Characterization Using X-ray Diffraction

XRD was employed to examine the crystallographic properties of THN, P-407, STA, HPMC, and CRMS-F15. The XRD 6100 (Shimadzu, Kyoto, Japan) instrument was used for this analysis. Diffractograms were generated covering a 2-theta range from 10 to 80 degrees, utilizing a step size of 0.02 degrees and a counting time of 0.30 s per step. The settings for the instrument comprised a voltage of 40 kV and a current of 30 mA. To ensure uniformity and facilitate random particle orientation, all samples were finely ground prior to the analysis [49]. Data analysis focused on peak position, intensity, and width to determine crystallographic properties of the sample, including phase identification and particle size [50].

#### 2.2.7. Characterization Using Scanning Electron Microscopy 

SEM was utilized to examine the surface morphology and microstructure of THN, P-407, STA, HPMC, CRMS-F15, and the branded drug. Imaging experiments were conducted using a JSM-6010PLUS/LA scanning electron microscope (JEOL Ltd., Tokyo, Japan), set to an accelerating voltage of 20 kilovolts (kV) for high-resolution imaging. Small quantities of each sample were mounted on specimen-holder stubs using double-sided adhesive carbon tape. To improve conductivity and facilitate accurate imaging, the samples underwent gold sputtering in a vacuum chamber using the Cressington 108 Auto Sputter Coater (Ted Pella Inc., Redding, CA, USA) [51].

#### 2.2.8. Characterization Using Fourier Transform Infrared 

FTIR was employed to analyze the molecular composition and identify functional groups in THN, P-407, STA, HPMC, and CRMS-F15. The IRAffinity-1 CE instrument (Shimadzu, Kyoto, Japan) was used for this analysis. It was calibrated to span a spectral range from 4000 cm^−1^ to 400 cm^−1^ wavenumbers, and data were collected from 45 scans to ensure the precise examination of molecular vibrations and absorptions. Data processing was conducted using IResolution software, version 1.60, facilitating a comprehensive analysis and extraction of detailed molecular information [52].

#### 2.2.9. Thermal Analysis via Differential Scanning Calorimetry 

Differential scanning calorimetry (DSC) analysis was conducted using a DSC-60 Plus instrument (Shimadzu, Kyoto, Japan) to study the thermal profiles of THN, P-407, STA, HPMC, and CRMS-F15. For this analysis, 3–5 mg of each sample was carefully weighed and placed on the sample pan under controlled conditions. The temperature range for the DSC scans was set from 25 °C to 350 °C at a heating rate of 10 °C per minute, and an inert atmosphere was maintained with a continuous flow of nitrogen at 100 mL/min [53]. The resulting thermograms were recorded and analyzed using Lab Solutions TA 60 software, focusing on evaluating the thermal properties of the drug powder, excipients, and the drug–excipient mixture, including aspects such as their melting points and thermal stability [51,54].

#### 2.2.10. Thermal Analysis via Thermogravimetric Analysis 

TGA was performed using a TGA-50 instrument (Shimadzu, Kyoto, Japan) to evaluate the thermal stability of THN, P-407, STA, HPMC, and CRMS-F15. Every sample, with a precise weight falling within the range of 10–15 mg, was meticulously weighed and then positioned in an alumina pan under controlled environmental conditions. Temperature scanning for each sample was conducted from 0 °C to 600 °C at a heating rate of 15 °C per minute, under a consistent nitrogen atmosphere flowing at 50 mL/min [53]. All acquired data were processed and interpreted using LabSolutions TA software [55]. 

#### 2.2.11. Stability under Varied Storage Temperatures and Humidity Levels

CRMS-F15 was prepared using the previously described procedure. Its stability was evaluated with one batch at different storage temperatures (8 °C, 20 °C, and 40 °C) with the humidity consistently maintained at 60%, and another batch at different humidity conditions (0%, 25%, 60%) while maintaining a constant temperature of 20 °C. For this, the CRMS-F15 capsules were stored in a stability chamber (Binder, Bohemia, NY, USA) set to these specific conditions. The formulation’s stability was determined by assessing in vitro drug release and drug content after 72 h [56]. A third batch was stored at a constant temperature of 20 °C and a humidity level of 25%. In vitro drug release and drug content were assessed at regular monthly intervals over a period of 3 months [57].

#### 2.2.12. Statistical Analyses

The mean values of the variables underwent analysis using a mixed ANOVA test to assess the presence of significant differences among the groups. Statistical significance was inferred for *p*-values less than 0.05. All statistical analyses were conducted utilizing Statistical Package for the Social Sciences (SPSS), version 26 [58].

## 3. Results

### 3.1. Calibration Curve and Drug Content

**Figure 2:**
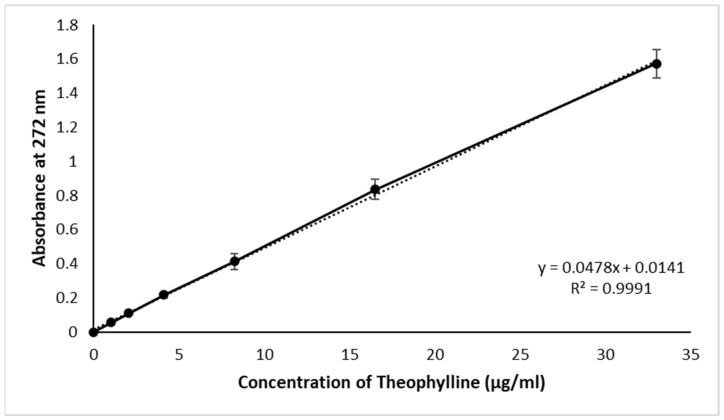
Calibration curve for theophylline detection via UV–visible spectrophotometry at 272 nm. The solid line represents the line of best fit, indicating the relationship between theophylline concentration and absorbance. The dotted lines show the standard deviation of the measurements at each concentration point, reflecting the variability of the data (average ± standard deviation; sample size = 3).

The calibration curve depicted in Figure 2 exhibits a high level of precision, as evidenced by its correlation coefficient (r^2^) of 0.9991. The high correlation coefficient (r^2^) of 0.9991, as shown in Figure 2, indicates the reliability of this calibration curve for the accurate determination of drug concentration in unknown samples. The drug content in CRMS-F15 was quantified as 300 ± 1.8 mg. This measurement aligns with the established criteria of the USP for drug content assays, confirming that the CRMS-F15 formulation adheres to these rigorous standards. This adherence to USP standards underscores the potential suitability of this formulation for further pharmaceutical applications [59].

### 3.2. In Vitro Drug Release 

This section presents the drug release profiles of the developed formulations and compares them to the THN drug without any added excipients. This comparison was designed to evaluate the effectiveness of the excipients in modulating drug release. Additionally, the release profile of the THN branded drug was assessed for its comparability with our developed formulations. The formulation demonstrating a release profile similar to that of the THN branded drug was then selected for further in vitro assessments across different pH environments (pH 1.2, 4.5, and 7.5).

#### 3.2.1. In Vitro Drug Release: First-Series Analysis

**Figure 3:**
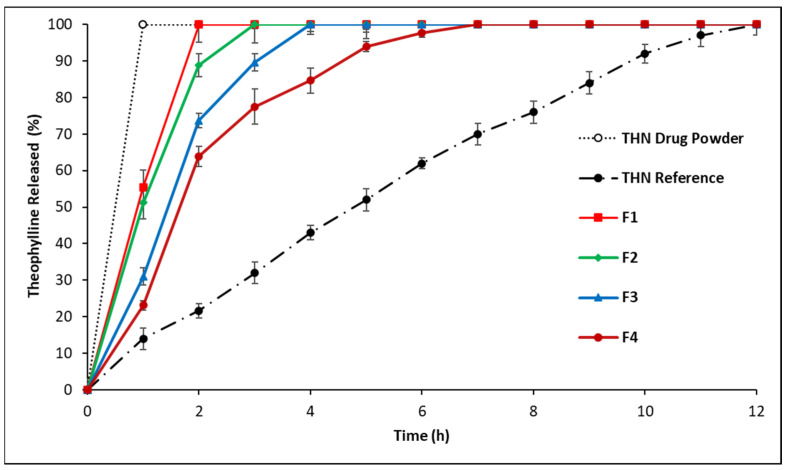
Release profiles of THN from polymer matrices with constant drug load and variable P-407 ratios, compared to the THN reference and THN drug powder over 12 h at 37 °C (average ± standard deviation; sample size = 3).

In Figure 3, the drug release profile of the branded THN drug, labelled as the THN reference, demonstrates a CR for up to 12 h. A significant variation in the drug release rate was observed among the first series of formulations (*p* < 0.05). 

The release rate decreased in the following order: THN branded drug > F4 > F3 > F2 > F1, which corresponds to the respective amounts of incorporated P-407: 70%, 60%, 50%, and 40%. An increase of 10% in the P-407 content resulted in at least a 1 h delay in drug release, indicating a clear correlation between the amount of P-407 and the decrease in release rate. Notably, formulation F4, containing 70% P-407, exhibited the most-extended controlled drug release within the first series, lasting up to 7 h. These findings underscore the significant role of P-407 in extending the drug release profile. 

#### 3.2.2. In Vitro Drug Release: Second-Series Analysis

**Figure 4:**
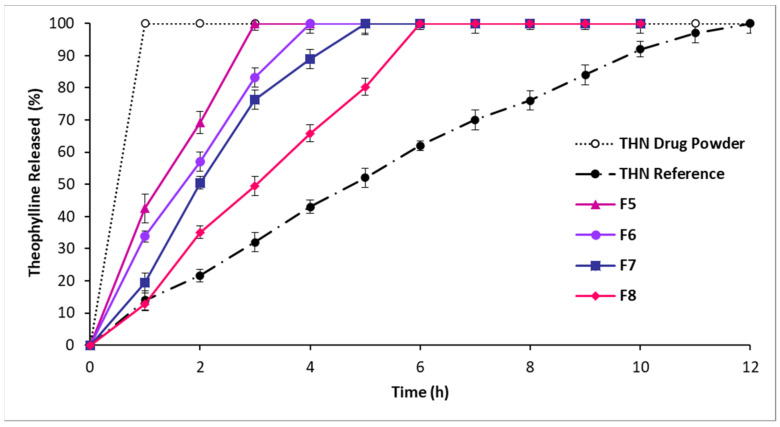
Release profiles of THN from polymer matrices with constant P-407 level and drug load and variable STA ratios, compared to the THN reference and THN drug powder over 12 h at 37 °C (average ± standard deviation; sample size = 3).

To assess the effect of STA, HPMC, and their combination on drug release, the formulations in the second, third, and fourth series contained a constant amount of P-407. In the second series, an increase in the incorporated amount of STA led to a delay in the drug release, as shown in Figure 4. 

The longest periods of controlled drug release were observed in the following order: F8 > F7 > F6 > F5, with STA incorporated amounts of 3%, 9%, 17%, and 25%, respectively. F8, containing 45% P-407 and 25% STA, demonstrated the longest period of controlled drug release, up to 6 h. 

#### 3.2.3. In Vitro Drug Release: Third-Series Analysis

**Figure 5:**
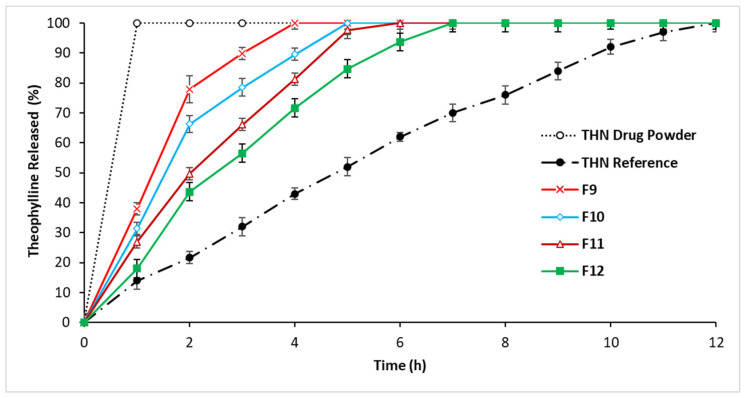
Release profiles of THN from polymer matrices with constant P-407 level and drug load and variable HPMC ratios, compared to the THN reference and THN drug powder over 12 h at 37 °C (average ± standard deviation; sample size = 3).

The substitution of STA with HPMC in the third series of formulations, as shown in Figure 5, effectively extended the drug release duration. An increase in HPMC concentration was directly associated with a longer delay in drug release. Notably, formulation F12 exhibited the most prolonged drug release control, lasting up to approximately 7 h. This was followed by formulations F11, F10, and F9, in that order. While the first three series demonstrated a significant impact of P-407, STA, and HPMC in prolonging drug release, the maximum duration they achieved was 7 h. 

#### 3.2.4. In Vitro Drug Release: Fourth-Series Analysis 

**Figure 6:**
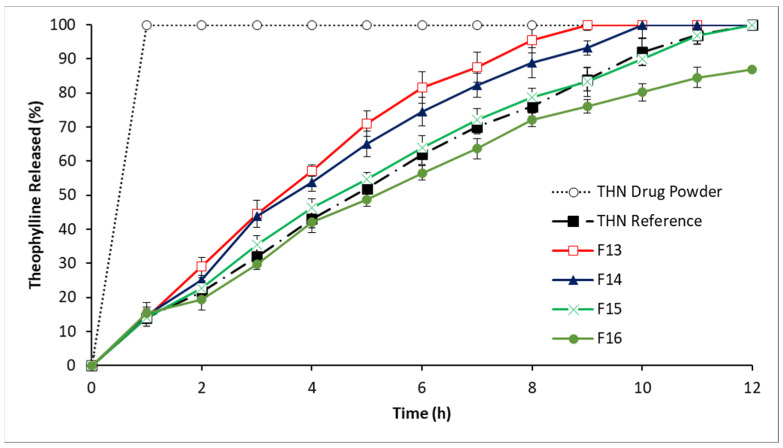
Release profiles of THN from polymer matrices with constant P-407 level and drug load and variable STA and HPMC ratios, compared to the THN reference and THN drug powder over 12 h at 37 °C (average ± standard deviation; sample size = 3).

Figure 6 presents the release profiles of THN from the matrix systems in Series 4, demonstrating the predominant influence of STA content on THN release from P-407/STA/HPMC matrix systems. An inverse relationship is observed: as the STA proportion increases, the percentage of THN release correspondingly decreases. For instance, the drug release level after 9 h varied across different compositions: it was 100% in the 20/20 HPMC/STA composition (F13), but was reduced to 93% in the 15/25 HPMC/STA composition (F14), 83% in the 10/30 HPMC/STA composition (F15), and 80% in the 5/35 HPMC/STA composition (F16). Notably, the release profile of F15 did not significantly differ (*p* > 0.05) from the reference THN product, maintaining THN release for up to 12 h. 

Furthermore, the results indicate a high degree of similarity between the test (F15) and reference dissolution profiles, as evidenced by an *f*2 similarity factor of 80, which is within the acceptable range of 50–100. This suggests that the test (F15) and reference profiles are closely matched. Additionally, the *f*1 dissimilarity factor of 3, which falls within the desirable range of 0–15, further confirms the minimal dissimilarity between these two profiles. Together, these factors demonstrate a strong alignment between the test formulation and the reference.

Consequently, CRMS-F15 was selected as the candidate formulation for further analysis and characterization.

#### 3.2.5. In Vitro Drug Release in Media of Different pH Levels (pH 1.2, 4.5, 7.5)

**Figure 7:**
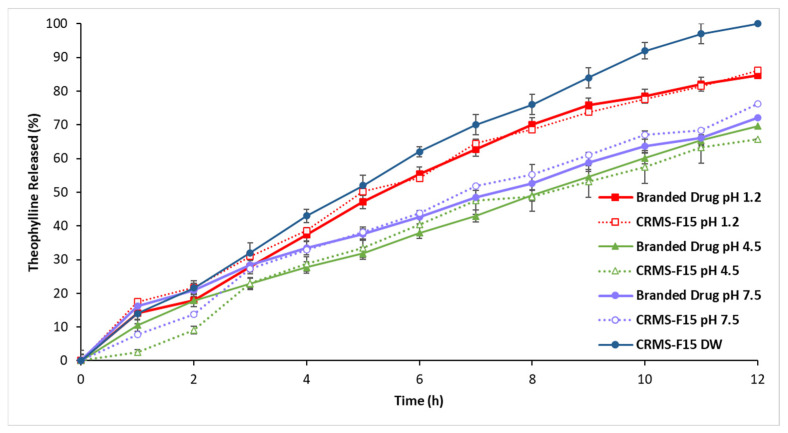
Release profiles of CRMS-F15 and the branded drug in media of different pH levels (1.2, 4.5, 7.5) over 12 h at 37 °C (average ± standard deviation; sample size = 3).

Figure 7 shows that the CRMS-F15 drug release profile did not significantly differ (*p* > 0.05) from the reference product in simulated physiological pH media at pH 1.2, 4.5, and 7.5. The matrix system was effective in controlling drug release for over 12 h. 

**Figure 8:**
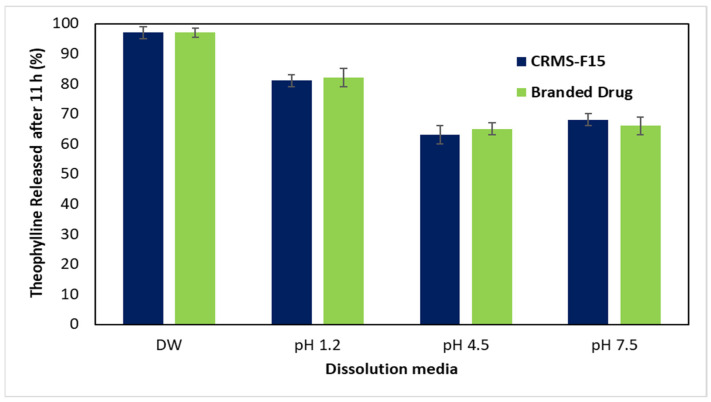
Release profiles of CRMS-F15 and the branded drug in media of different pH levels (1.2, 4.5, 7.5) at 11 h at 37 °C (average ± standard deviation; sample size = 3).

Figure 8 further illustrates the pH-dependent drug release from both CRMS-F15 and the reference product after 11 h. The highest drug release rate was observed at the water pH level, followed by pH 1.2, then pH 7.5, and it was lowest at pH 4.5, with only minor variations noted. The drug release rate from CRMS-F15 at 11 h was 97% in DW and 81%, 63%, and 68% at pH 1.2, pH 4.5, and pH 7.5, respectively.

### 3.3. Kinetic Release Model Evaluation 

The statistical analysis comparing the release kinetic model results of CRMS-F15 to those of the branded drug, as presented in Table 5, revealed no remarkable difference (*p* > 0.05). Among the evaluated models, both the zero-order and Hixson–Crowell models exhibited the highest goodness of fit, with $r^{2}$ values of 0.9774 and 0.9976, respectively. These results suggest that these models accurately described the release behavior of our drug formulation. The Higuchi model also demonstrated a relatively high correlation, with a coefficient of 0.9702. Conversely, the first-order and Korsmeyer–Peppas models showed lower $r^{2}$ values, indicating their limited suitability for describing the release kinetics of this formulation.

### 3.4. X-ray Diffraction 

**Figure 9:**
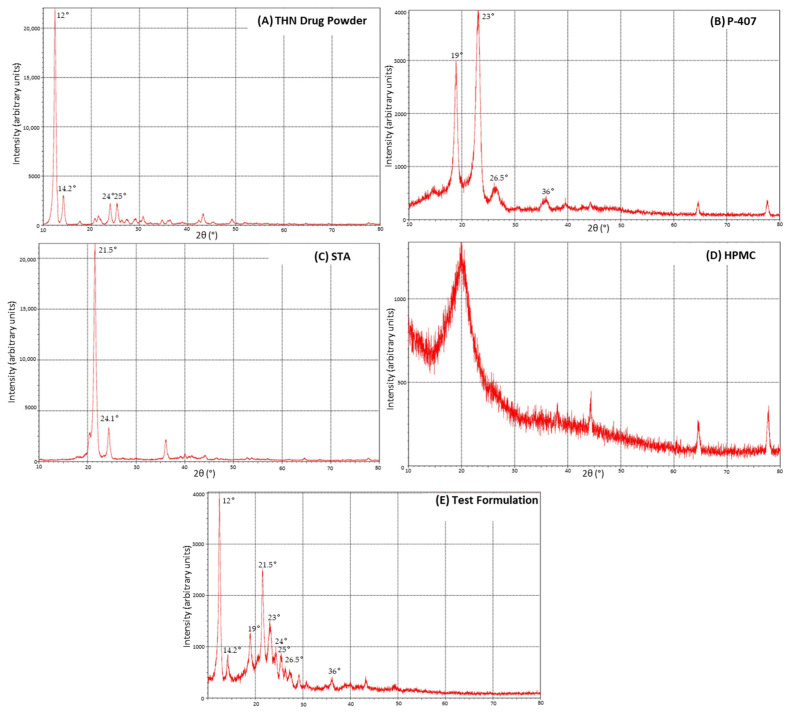
X-ray diffraction patterns of individual components: (**A**) theophylline, (**B**) poloxamer 407, (**C**) stearyl alcohol, (**D**) hydroxypropyl methylcellulose, and (**E**) the test formulation.

Figure 9 depicts the diffraction patterns for THN, P-407, STA, HPMC, and CRMS-F15. The diffractogram of THN featured several sharp peaks, with the most prominent at around 12° (2θ), and other less intense peaks at 2θ angles of 14.2, 24, and 25.3. This finding aligns with previous research demonstrating similar XRD patterns for THN (Figure 9A) [60]. 

P-407 exhibited sharp peaks indicative of a semi-crystalline structure, with the most intense peaks approximately at 19° and 23° (2θ) in addition to wider peaks 26.5° and 36° (2θ) (Figure 9B) [61,62]. The XRD pattern of STA showed an exceptionally intense peak at about 21.5° (2θ), with a less intense peak at 24.1° (2θ); these sharp peaks indicate a high degree of crystallinity in STA (Figure 9C) [63]. In contrast, HPMC presented an amorphous halo, lacking discernible crystalline peaks, except for a broad characteristic peak across the 15–25° (2θ) range, which is typical for many polysaccharides (Figure 9D) [64,65]. The XRD peak patterns of CRMS-F15, as illustrated in Figure 9E, showed peak positions similar to those of the drug molecule and the excipients used. However, a notable reduction in peak intensity was observed, particularly for the peaks associated with the drug molecule.

### 3.5. Scanning Electron Microscopy 

**Figure 10:**
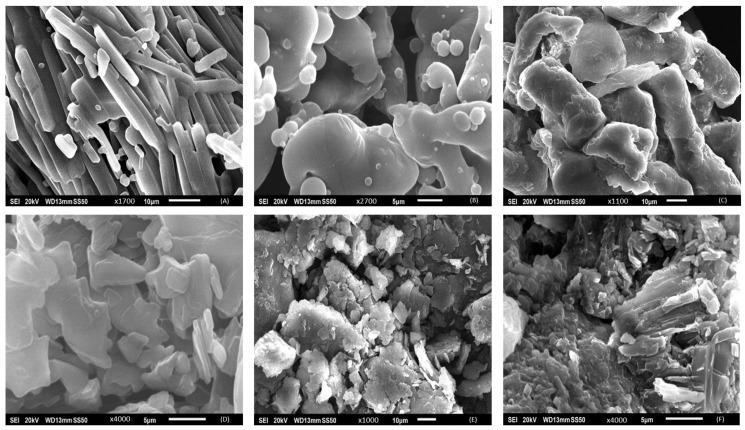
Scanning electron microscopy analysis of components and formulations: (**A**) theophylline, (**B**) poloxamer 407, (**C**) hydroxypropyl methylcellulose, (**D**) stearyl alcohol, (**E**) the test formulation, and (**F**) the THN reference.

SEM images, as shown in Figure 10, provided detailed morphological insights into the various studied materials, including THN, P-407, STA, HPMC, CRMS-F15, and the branded drug. The SEM micrograph of THN particularly highlights a distinctly crystalline structure (Figure 10A). Most particles appear rod-like in shape, with uniform surfaces and sharp edges, indicative of significant crystallinity. The dimensions of these crystalline structures vary, with some particles measuring over 10 µm in length while maintaining a width of less than 10 µm [66]. 

Contrastingly, SEM imagery showed the P-407 particles to be spherical with predominantly smooth surfaces, although some exhibit minor surface irregularities and are interspersed with smaller spheres (Figure 10B) [61]. The HPMC samples, however, significantly differed, displaying an assortment of irregular, non-crystalline structures. These are characterized by a coarse, heterogeneous surface texture, indicating an agglomerated state (Figure 10C). The SEM images of STA reveal a compact, stratified arrangement, primarily flake-like in morphology. The surfaces of these laminar flakes are noticeably rough, with many visible edges and facets. Their sizes vary, with some extending several micrometers in length (Figure 10D).

The SEM examination of CRMS-F15, as shown in Figure 10E, reveals a composite microstructure. This arrangement is characterized by a uniform mixture of constituents, suggesting an integrated composite. The matrix of this formulation displays a slightly uneven texture, which is similar to that of the branded drug, depicted in Figure 10F.

### 3.6. Fourier Transform Infrared 

**Figure 11:**
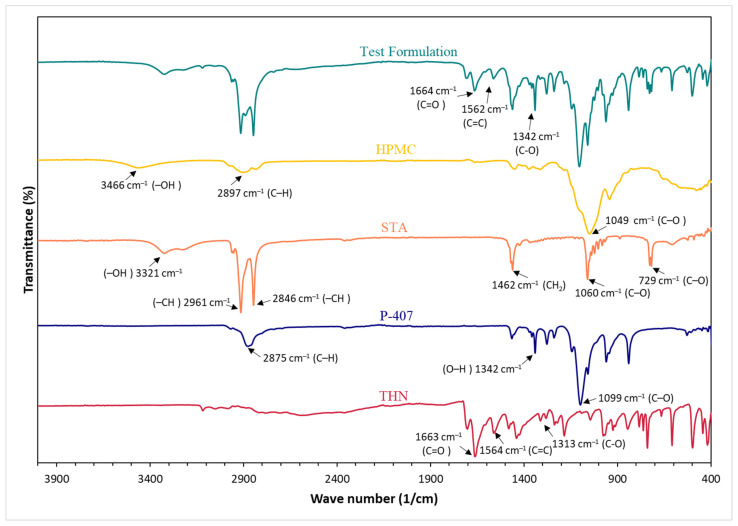
Fourier transform infrared spectra of theophylline powder, poloxamer 407, hydroxypropyl methylcellulose, stearyl alcohol, and CRMS-F15.

The spectral profiles generated via FTIR for the THN powder, P-407, STA, HPMC, and CRMS-F15 are illustrated in Figure 11. The primary functional groups of THN, associated with various vibrational modes, exhibited clear signals at 1313 cm^−1^, 1663 cm^−1^, and 1564 cm^−1^, representing the stretching of C-O, amide C=O, and aromatic C=C bonds, respectively. These findings were consistent with those of a prior study [67].

The IR spectra of P-407 exhibited distinctive peaks, including a prominent absorption at 2875 cm^−1^ corresponding to aliphatic C–H stretching vibrations. Furthermore, these spectra featured a well-defined peak at 1342.46 cm^−1^, attributed to O–H bending, and a sharp absorption at 1099 cm^−1^, indicating C–O stretching [68].

The STA spectra displayed moderate-intensity peaks at 2846 and 2961 cm^−1^, which are indicative of aliphatic –CH stretching vibrations. The absorption peak at 3321 cm^−1^ corresponds to –OH stretching vibrations, while peaks at 1462 cm^−1^ and 1060 cm^−1^ represent –CH_2_ and C–O stretching, respectively. An additional peak associated with C–O stretching was observed at 729 cm^−1^ [69].

Regarding HPMC, the absorption peak at 3466 cm^−1^ is attributed to the stretching frequency of the -OH group. Additional stretching vibration bands associated with C-H and C-O were detected at 2897 cm^−1^ and 1049 cm^−1^, respectively. 

The main peaks of THN remained evident in the IR spectra of CRMS-F15, exhibiting only negligible shifts in wavenumber. For instance, peaks at 1342 cm^−1^ (C-O stretch), 1664 cm^−1^ (C=O stretching amide), and 1562 cm^−1^ (C=C stretching aromatic) were consistent in both spectra.

### 3.7. Differential Scanning Calorimetry 

**Figure 12:**
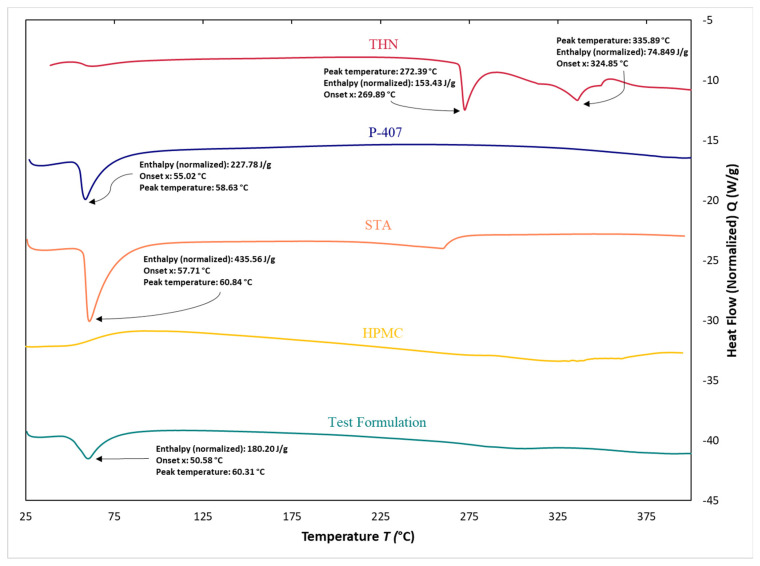
Differential scanning calorimetry results of individual components: theophylline, poloxamer 407, stearyl alcohol, and hydroxypropyl methylcellulose.

Figure 12 presents the thermal profiles from DSC analyses of the pure THN, P-407, STA, and HPMC. The DSC thermogram of THN shows two prominent endothermic peaks. The first peak initiated at an onset temperature (*T*_onset_) of 269.89 °C, peaked at 272.39 °C, and exhibited a heat absorption of 153.43 J/g. The second peak began at a *T*_onset_ of 324.85 °C, reached a peak temperature of 335.89 °C, and had an enthalpy of 74.849 J/g. For P-407, DSC analysis revealed a significant endothermic peak. This thermal event began at a *T*_onset_ of 55.02 °C, peaked at 58.63 °C, and showed an enthalpy flow of 227.78 J/g.

The DSC thermogram for STA displays a prominent endothermic peak, beginning at a *T*_onset_ of 57.71 °C with an enthalpy of 435.56 J/g, and peaking at 60.84 °C. In contrast, no characteristic peak was observed for HPMC. 

**Figure 13:**
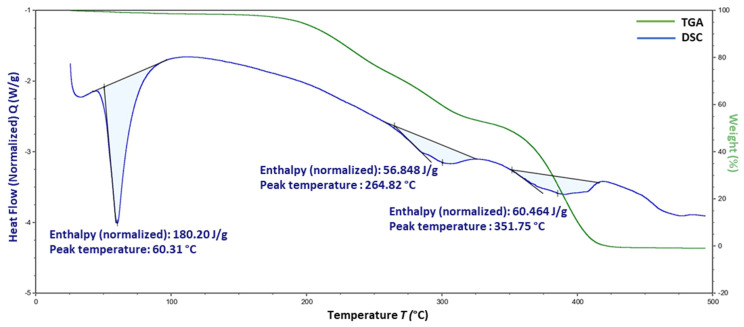
Differential scanning calorimetry and thermogravimetric analysis of CRMS-F15.

In the DSC analysis of the CRMS-F15, three distinct endothermic peaks were observed, as shown in Figure 13. The initial peak had an enthalpy of 180.20 J/g and a peak temperature of 60.31 °C. This was followed by a secondary peak with a peak temperature at 264.82 °C, exhibiting an enthalpy of 56.848 J/g. The tertiary peak had a peak temperature of 351.75 °C with an enthalpy of 60.464 J/g. 

These peaks indicate the thermal behavior of the incorporated excipients and the drug molecule. Notably, the enthalpy values, represented by the area under the curve of each peak, showed a decrease when compared to those of the individual constituents. The peak at 60.31 °C is hypothesized to arise from the combined melting points of P-407 and STA. The peak at 264.82 °C likely represents the melting point of THN; additionally, the peak at 351.75 °C is assumed to be attributed to degradation of THN.

### 3.8. Thermogravimetric Analysis 

**Figure 14:**
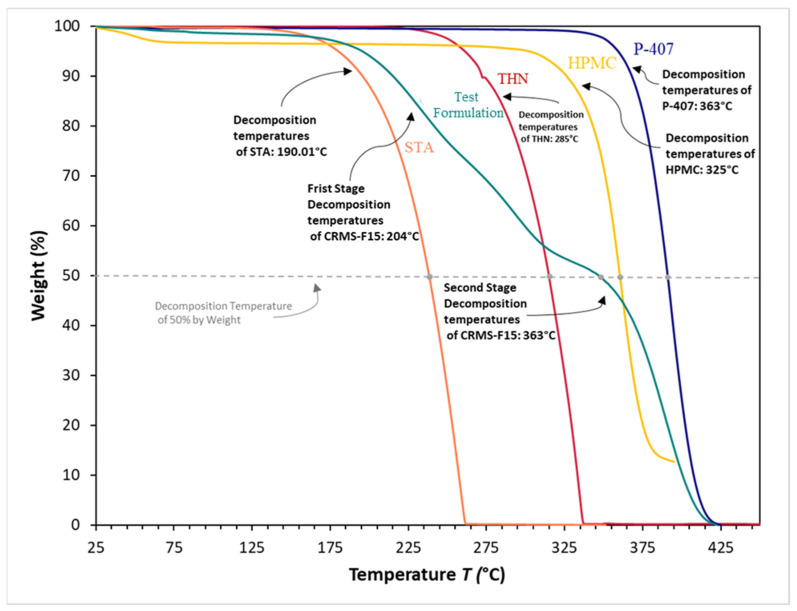
Thermogravimetric analysis of individual components: theophylline, poloxamer 407, hydroxypropyl methylcellulose, stearyl alcohol, and CRMS-F15.

The TGA results showed that THN, as well as all the materials used in developing the matrix system, underwent a single stage of thermal decomposition and exhibited relatively high decomposition temperatures, as shown in Figure 14. For THN, the decomposition began at 285 °C, resulting in a residual weight of 84.296%. The decomposition of THN completed at 336.55 °C, leaving a final weight of 1.014%. The recorded decomposition temperatures for P-407, STA, and HPMC were 363 °C, 190.01 °C, and 325 °C, respectively. In contrast, the thermal decomposition of CRMS-F15 occurred in two stages: the first stage at 204 °C with a remaining weight of 94.07%, followed by the second stage at 363 °C, with a remaining weight of 44.27%. 

The temperatures required to induce a 50% weight loss in the samples, ranked from highest to lowest, were as follows: P-407 at 390.97 °C, HPMC at 360.38 °C, CRMS-F15 at 346.91 °C, THN at 314.93 °C, and STA at 238.27 °C. 

### 3.9. Stability Assessment 

**Figure 15:**
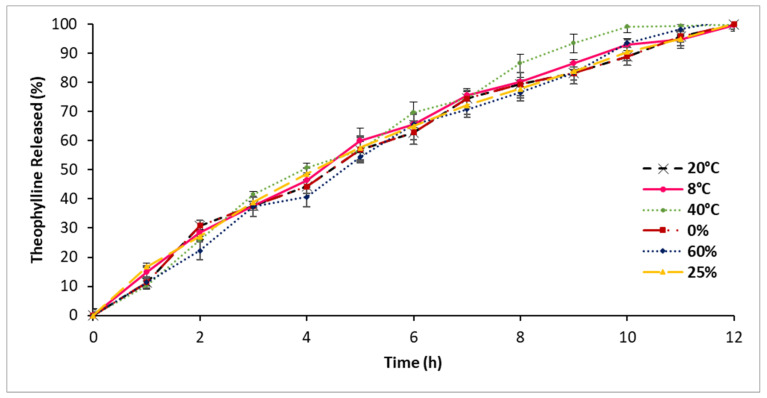
Release profiles of the test formulation (CRMS-F15) after 72 h storage at different temperatures (8 °C, 20 °C, 40 °C) with consistent humidity (60%) and at different humidity levels (0%, 25%, 60%) with constant temperature (20 °C) (average ± standard deviation; sample size = 3).

Figure 15 demonstrates the release profile of CRMS-F15 during a 72 h storage period under varying temperatures (8 °C, 20 °C, 40 °C) with consistent humidity (60%). These results indicate that there were no significant differences in the drug release profiles of CRMS-F15 when stored under different temperatures (*p* > 0.05). A slight increase in drug release was observed for the formulation stored at the highest temperature of 40 °C; however, this increase remained within permissible limits.

Figure 15 also illustrates the drug release profile of CRMS-F15 stored under different humidity conditions (0%, 25%, 60%) under a constant temperature, compared to the release profile of a fresh CRMS-F15 sample. There was no significant difference in drug release before 72 h across these various storage conditions (*p* < 0.05).

Regarding drug content, no significant differences were observed when stored under varying temperature or humidity conditions (*p* > 0.05), as shown in Table 6. The minor variations in drug content following these storage conditions remained within the permissible limits [59].

**Figure 16:**
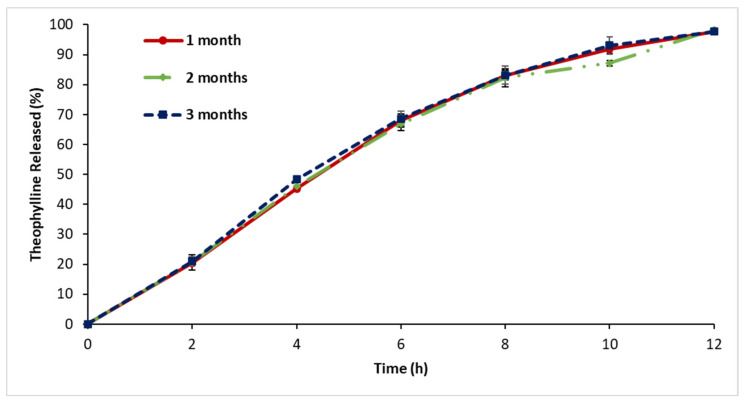
Release profiles of test formulation (CRMS-F15) stored at 20 °C with 25% humidity for 1, 2, and 3 months (average ± standard deviation, sample size = 3).

Figure 16 illustrates the in vitro release of CRMS-F15 when stored at 20 °C and 25% relative humidity, with assessments conducted after storage durations of 1, 2, and 3 months. The release profiles showed minimal variation across the different storage periods when evaluated at specified time points over a 12 h duration. Similarly, the drug content over the full three-month period was within the range of 294 to 302 ± 3.5.

## 4. Discussion

### 4.1. In Vitro Drug Release

The observed positive correlation between delayed drug release and the increased concentration of the excipients utilized (P-407, HPMC, and STA) in the formulation of the new CRMS indicates the efficacy of these excipients in controlling the release of THN. Key determinants in the polymer’s capacity to modulate drug release include the concentration, the HLB value, the chemical structure of the polymer, and its mechanical strength [70,71,72,73]. In this section, we discuss the results obtained through our in vitro drug release assessments.

#### 4.1.1. In Vitro Drug Release: First-Series Analysis 

The controlled drug release observed in the initial series can be primarily attributed to the amphiphilic nature of P-407. This polymer effectively retains the drug molecules, facilitating their gradual release. The mechanism behind this CR involves the continuous swelling of the polymer carrier upon exposure to liquid media. This swelling process forms a gel-like barrier around the formulation. Concurrently or subsequently, the polymer undergoes dissolution, further contributing to the CR dynamics [56,74]. Gel formation within this matrix system is initiated by the hydration and subsequent swelling of the hydrophilic chains of P-407, accompanied by the aggregation of hydrophobic components to minimize their contact with the aqueous media. This structure, achieved by dispersing the drug throughout the matrix system, allows the hydrophobic chains to act as a barrier against water diffusion. Consequently, this barrier significantly restricts the diffusion of the drug, leading to a controlled and delayed release [75]. 

The dynamic interaction between hydrophilic and hydrophobic components is crucial in controlling drug release kinetics. As the P-407 concentration in the formulation increases, the distance between polymeric networks decreases. This reduced spacing enhances cross-linking within the polymer, thereby strengthening the gel structure and increasing viscosity. As a result, a more pronounced delay in drug release is observed. Additionally, the enhanced hydrophobic chain effect further limits water diffusion into the matrix system, reinforcing the CR mechanism [56].

These findings are consistent with previous research, as outlined in the literature section, which demonstrates that an increased concentration of P-407 in formulations consistently leads to delayed drug release across various types of dosage forms [33,56,76]. For instance, Zhang et al. developed a topical CR formulation containing ceftiofur for foot infections. Their results indicated that higher concentrations of P-407 in the gel formulation led to delayed drug release. Specifically, the formulation with 25% P-407 released 80% of the drug within 5 h, compared to a 70% release from the formulation with 30% poloxamer and only a 40% release from the formulation with 35% P-407 [33]. 

Although in our study, the sole use of P-407 successfully delayed drug release up to 7 h, we presume that P-407 alone may not be sufficient to achieve a 12 h delay. This limitation is likely due to its limited mechanical strength, which makes it more susceptible to dissolution in water [77,78]. Consequently, as detailed in the next three sections of our study, we proceeded to incorporate various excipients and ratios alongside P-407 to evaluate their effectiveness in sustaining drug release.

#### 4.1.2. In Vitro Drug Release: Second-Series Analysis

In the second experiment series, the concentration of P-407 was maintained constant to specifically assess the influence of STA on drug release delay. The observed delay in drug release can be attributed to the lipophilic nature, stiffening effect, and viscosity enhancement properties of STA, as referenced in [79,80]. 

As the proportion of STA increases in our formulation, it not only enhances the matrix system’s hydrophobic nature but also amplifies intermolecular interactions. These synergistic effects collectively strengthen the matrix’s mechanical properties, thereby more effectively limiting water diffusion into the formulation and further delaying drug release. Our study aligns with the research conducted by Cao et al., who prepared a sustained-release tablet using STA, stearic acid, and carnauba wax for a highly water-soluble model drug. Their in vitro assessment revealed that increasing the proportion of STA further delayed drug release [79].

#### 4.1.3. In Vitro Drug Release: Third-Series Analysis

The amount of P-407 was kept consistent throughout Series 3 in order to evaluate the impact of HPMC on controlling drug release. The decrease in drug release, corresponding to the increase in HPMC concentration, is presumed to be associated with several factors. The entanglement of polymer chains between HPMC and P-407 creates a complex and interconnected network structure, enhancing resistance to flow and increasing the thickness of the gel. Several studies have supported these results, as mentioned earlier in the literature section [33,48,56,81,82].

#### 4.1.4. In Vitro Drug Release: Fourth-Series Analysis 

Series 4 is assumed to exhibit prolonged drug release compared to the first three series due to the stronger intermolecular interactions that occur when P-407, STA, and HPMC are combined. This contrasts with the separate interactions of HPMC or STA with P-407. As the intermolecular interactions intensify, they lead to gel shrinkage and an increase in viscosity, consequently slowing down the drug release. Furthermore, this robust interaction fortifies the matrix system against degradation and dissolution, thus further delaying drug release [83,84].

The CR of the medication predominantly depends on the gel formed by P-407, which regulates drug release. The incorporation of HPMC enhances the gel’s viscosity through the mechanism described earlier. Additionally, the lipophilic and stiffening properties of STA impede water diffusion into the formulation, further reducing drug dissolution [70].

Based on these findings, the recommended concentrations of STA and HPMC for extending drug release up to 12 h are 30% and 10%, respectively, in addition to 30% P407. Consequently, CRMS-F15 has been identified as the candidate formulation. Notably, CRMS-F15 exhibited a release profile similar to that of the FDA-approved branded THN product, according to the results of a fit factor analysis between the tested formulation and the reference product, suggesting potential interchangeability between this formulation and the branded product.

#### 4.1.5. In Vitro Drug Release in Media of Different pH Levels (pH 1.2, 4.5, 7.5)

The variation in drug release among different pH levels is not influenced by the excipients, as they are non-ionic and function consistently across various pH levels [56,85]. However, THN’s solubility varies with changes in pH due to its amphoteric nature, where it can act as both an acid and a base. This is attributed to its functional groups: the nitrogen (N), with a lone pair of electrons, acts as a basic site by accepting protons (H+), while the NH group behaves as an acidic site by donating H+ to the surrounding media. With a pKa of 8.87, THN is 50% ionized at this pH, with ionization increasing as the pH rises. Conversely, at a pKb of 11.5, the drug’s ionization increases below pH 2.5 [86].

Consequently, when the pH ranges from 2.5 to 8.87, THN undergoes minimal or no ionization, resulting in reduced drug solubility. This leads to the lowest release rate of THN at pH 4.5. However, at pH 1.2, well below 2.5, increased ionization occurs, facilitating the release of over 50% of the drug and thus yielding a higher release rate. Given THN’s pKa of 8.87, the drug undergoes limited ionization at pH 7.5. As a result, THN demonstrates a higher release rate at pH 7.5 compared to pH 4.5 and pH levels below 1.2 [56,85]. The lack of significant variations in the release profiles across different pH media, when compared to the reference product, indicates a similar functionality and stability of the new delivery system in relation to the reference product [56].

### 4.2. Release Kinetic Model Evaluation 

In our exploration of the release kinetics of the new formulation, the Hixson–Crowell and zero-order kinetic models emerged as the primary mechanisms driving drug release. The Hixson–Crowell model indicates that drug release is predominantly controlled by erosion processes, reflecting the formulation’s design where drug release is regulated through gradual erosion or dissolution of the delivery matrix. Simultaneously, the compliance with zero-order kinetics, as demonstrated by our data, underscores a mechanism that sustains a steady drug release rate over time. This consistent rate, unaffected by the drug concentration in the formulation, is essential for ensuring uniform therapeutic efficacy [31,87].

### 4.3. X-ray Diffraction 

The alignment of peak positions in CRMS-F15’s XRD pattern with those of the individual excipients and active ingredients suggests that all materials, including the drug molecule, maintained their crystalline structure, indicating their compatibility. This also implies that no degradation of the active ingredient occurred during the fusion process [88].

However, the diminished peak intensity in the XRD pattern of CRMS-F15, relative to the corresponding peaks of each individual ingredient, implies a dispersion of the drug within the matrix system. This dispersion likely leads to a reduced number of particles available to reflect the X-rays when compared to the individual materials in isolation [64,65,89].

Our findings align with those reported by Ellakwa et al., where an immediate-release tablet formulated with poloxamer 407 was used to deliver mosapride citrate. Their XRD analysis showed a reduction in the peak intensity of the active ingredient, suggesting its dispersion within the carrier matrix [88]. Similar results were also demonstrated by El-Badry et al. [62].

### 4.4. Scanning Electron Microscopy 

Our SEM observations of CRMS-F15 support the XRD findings, showing the dispersion of drug molecules within the matrix system. It is clearly evident that the SEM images of CRMS-F15 display a variety of irregular morphological features in the molecules, similar to those observed in the branded drug, which also demonstrates an irregular molecular morphology [90]. 

### 4.5. Fourier Transform Infrared 

The presence of the peak corresponding to THN’s functional group in CRMS-F15 is evidence of successful drug dispersion within the matrix system. This indicates that the drug molecules remain intact, exhibiting no signs of degradation or adverse interactions with the polymers [67,78,89].

Comparable findings were reported by Ashames et al. in their research on developing a novel polymeric matrix system. Notably, in their study, distinct peaks corresponding to the functional groups of the active ingredient were observed in the drug-loaded matrix system, reinforcing the consistency of these outcomes in polymer-based drug delivery research [89]. Similar findings were also reported in studies conducted by Ice et al. [78]. In another study, researchers formulated a CR system employing carboxymethyl chitosan for the regulated release of THN. The FTIR results of this study were in agreement with our findings, underscoring the preservation of THN’s integrity. This was evidenced by the lack of significant spectral variations in the final product compared to the pure drug molecule [67].

### 4.6. Differential Scanning Calorimetry 

In the results of the DSC analysis of THN, the endothermic peak at 272.39 °C is assumed to be the melting point of THN. This assumption is corroborated by previous studies that have reported similar melting points for THN at 272.6 °C [91] and 269.7 °C [92], confirming the alignment of our results with the existing literature.

Additionally, the sharpness of the observed peak could indicate a high degree of crystallinity in THN, a finding that is supported by our XRD results. Crystalline materials, known for their more-ordered structures, typically undergo phase transitions, such as melting, within a narrower temperature range. This characteristic leads to the formation of sharper peaks in DSC [42].

The endothermic peaks observed for P-407 and STA are presumed to correspond to their respective melting points, aligning with findings from previous studies. EL-Badry et al., in their investigation of P-407 as a hydrophilic delivery system for nimesulide, observed a distinct endothermic peak at 55 °C in their DSC results, attributed to the melting of P-407. Similarly, other studies have identified the melting point of STA to be 34 °C [63]. The thermal behavior of HPMC observed in our study is consistent with that reported in prior research [93]. 

In CRMS-F15, the observed decrease in heat flow is presumed to be attributed to the dilution effect of the excipients and the drug molecule. This effect results from the reduced concentration of each component per gram in the matrix system compared to their concentrations in individual states. Due to this dilution, a lesser amount of the material undergoes melting, leading to a decreased area under the curve. Such an observation may also indicate successful dispersion of the drug within the matrix system [89]. 

Supporting this hypothesis, Bouriche et al. developed a metformin-loaded polylactic-acid microparticle. Their DSC assessment of this formulation revealed a marked reduction in the peak corresponding to metformin. This finding was interpreted as indicative of successful drug dispersion within the polymer matrix system [94].

### 4.7. Thermogravimetric Analysis 

The decomposition temperature of CRMS-F15 exhibits two stages, indicating distinct thermal behaviors. The first stage, beginning at 204 °C, suggests the initial breakdown of the more thermally sensitive components, including STA and THN in the formulation. The second stage, occurring at 363 °C, indicates the decomposition of the more thermally resistant components, which are P-407 and HPMC [95].

Our findings are corroborated by various studies. For instance, Mikrani et al. developed a solid dispersion formulation for low-solubility medications, particularly BCS Class II drugs, using P-407 and HPMC. TGA showed their formulation to have a thermal stability profile almost identical to that of our candidate formulation, with the initial stage of decomposition starting beyond 198 °C [96]. 

The TGA profile of our formulation revealed the emergence of a combined thermal behavior of the used excipients and the drug. The temperature required to induce a 50% weight loss in CRMS-F15 appeared mid-range among all the used components. This is indicative of the successful integration of the drug within the matrix system [89,97].

### 4.8. Stability Assessment

In terms of stability assessment, CRMS-F15 exhibited remarkable physical and chemical stability. This was evidenced by the consistent drug release profile and drug content following storage under various temperature and humidity conditions. Similarly, the consistent release rate and drug content over long-term storage conditions indicate the formulation’s stability. 

## 5. Conclusions 

In conclusion, this study has successfully developed a new dosage form of a CRMS capsule for THN, incorporating P-407, HPMC, and STA. The research demonstrates a significant positive correlation between the concentrations of these excipients and the extent of drug release delay, confirming their effectiveness in controlling drug release. This newly developed CRMS offers a viable alternative for other BCS Class I drugs, particularly due to its release profile, which aligns closely with the FDA-approved branded THN drug. Its ability to control a 12 h release, combined with its proven stability and compatibility, establishes it as a significant advancement in pharmaceutical drug delivery systems.

## Figures and Tables

**Table 1:** Percentage compositions of THN and P-407 in the first-series controlled-release matrix system formulations.

Formulations	THN (%)	P-407 (%)	Total Weight (mg)
F1	60	40	500
F2	50	50	600
F3	40	60	750
F4	30	70	1000

**Table 2:** Percentage compositions of THN, P-407, and STA in the second series controlled-release matrix system formulations.

Formulations	THN (%)	P-407 (%)	STA (%)	Total Weight (mg)
F5	39	58	3	775
F6	36	55	9	825
F7	33	50	17	900
F8	30	45	25	1000

**Table 3:** Percentage compositions of THN, P-407, and HPMC in the third-series controlled-release matrix system formulations.

Formulations	THN (%)	P-407 (%)	HPMC (%)	Total Weight (mg)
F9	39	58	3	775
F10	36	55	9	825
F11	33	50	17	900
F12	30	45	25	1000

**Table 4:** Percentage compositions of THN, P-407, STA, and HPMC in the fourth-series controlled-release matrix system formulations.

Formulations	THN (%)	P-407 (%)	HPMC (%)	STA (%)	Total Weight (mg)
F13	30	30	20	20	1000
F14	30	30	15	25	1000
F15	30	30	10	30	1000
F16	30	30	5	35	1000

**Table 5:** Coefficient of determination (r^2^) results for CRMS-F15 and the branded drug across various release kinetic models.

Model Name	$r^{2}$ of F15	$r^{2}$ of Reference Product
Zero-order model	0.9774	0.9863
First-order model	0.8457	0.8466
Hixson–Crowell model	0.9976	0.9956
Higuchi model	0.9702	0.9600
Korsmeyer–Peppas model	0.6348	0.6265

**Table 6:** Drug content in formulation F15 after 72 h storage under varied temperature and humidity conditions for stability assessment.

Storage Temperature (°C)	Storage Humidity (%)	Drug Content (mg)
20	60	300.00 ± 1.70
08	60	299.14 ± 0.86
40	60	298.28 ± 1.90
20	00	300.00 ± 1.72
20	25	303.45 ± 1.71
20	60	300.52 ± 0.52

## Data Availability

Data are contained within the article.
